# Predose and Postdose Blood Gene Expression Profiles Identify the Individuals Susceptible to Acetaminophen-Induced Liver Injury in Rats

**DOI:** 10.1371/journal.pone.0141750

**Published:** 2015-10-29

**Authors:** Xiaoyan Lu, Bin Hu, Jie Zheng, Cai Ji, Xiaohui Fan, Yue Gao

**Affiliations:** 1 Pharmaceutical Informatics Institute, College of Pharmaceutical Sciences, Zhejiang University, Hangzhou, Zhejiang, China; 2 Department of Pharmacology and Toxicology, Beijing Institute of Radiation Medicine, Beijing, China; UFMG, BRAZIL

## Abstract

The extent of drug-induced liver injury (DILI) can vary greatly between different individuals. Thus, it is crucial to identify susceptible population to DILI. The aim of this study was to determine whether transcriptomics analysis of predose and postdose rat blood would allow prediction of susceptible individuals to DILI using the widely applied analgesic acetaminophen (APAP) as a model drug. Based on ranking in alanine aminotransferase levels, five most susceptible and five most resistant rats were identified as two sub-groups after APAP treatment. Predose and postdose gene expression profiles of blood samples from these rats were determined by microarray analysis. The expression of 158 genes innately differed in the susceptible rats from the resistant rats in predose data. In order to identify more reliable biomarkers related to drug responses for detecting individuals susceptibility to APAP-induced liver injury (AILI), the changes of these genes' expression posterior to APAP treatment were detected. Through the further screening method based on the trends of gene expression between the two sub-groups before and after drug treatment, 10 genes were identified as potential predose biomarkers to distinguish between the susceptible and resistant rats. Among them, four genes, *Incenp*, *Rpgrip1*, *Sbf1*, and *Mmp12*, were found to be reproducibly in real-time PCR with an independent set of animals. They were all innately higher expressed in resistant rats to AILI, which are closely related to cell proliferation and tissue repair functions. It indicated that rats with higher ability of cell proliferation and tissue repair prior to drug treatment might be more resistant to AILI. In this study, we demonstrated that combination of predose and postdose gene expression profiles in blood might identify the drug related inter-individual variation in DILI, which is a novel and important methodology for identifying susceptible population to DILI.

## Introduction

Drug-induced liver injury (DILI) is the main adverse drug reaction and often life-threatening. It is also a leading cause of drugs that never reach the market and being withdrawn after product launch. The relatively low incidence and idiosyncratic nature of most cases of DILI suggest that susceptibility to this injury might be determined by multiple genetic and acquired environmental factors [1]. Pharmacogenomics, which aims to define the genetic determinants of drug response, has been widely recommended as a potential approach of individualized drug therapy to guide therapeutic strategies toward a better safety and efficacy profile [2]. The principal goals of pharmacogenomics research on DILI are an elucidation of hepatotoxic mechanisms and the prediction of DILI in individuals [3]. Nevertheless, acquired environmental factors play an important role in individual metabolic phenotypes, modulating drug metabolism, efficacy, and toxicity, such as age, sex, ethnicity, nutritional status, gut bacterial activities, disease, and other drug use [4]. Such environmental complications will be limited the usefulness of prediction of drug-induced responses that are based only on genomic differences [4–6].

Recognizing this important limitation of pharmacogenomics and the potential of acquired environmental factors altering gene expressions [4], predose gene expression profiles have recently been used to predict a subject’s response to potential drug intervention. Yun et al. have recently devised a novel method that examines the innate gene expressions of biofluids and biopsy tissues prior to drug treatment [7–10], which might identify susceptible population to DILI. This method has a major advantage as the fact that the derived gene expression profiles are sensitive to both genomic and environmental influences. Moreover, a further crucial advantage of this method is its openness to finding unexpected biomarkers as the global gene expression is quantified simultaneously without prespecification of what those genes should be. However, one critical problem existed in this method is that a number of inter-individual variation in predose gene expression data before drug administration are not associated with drug responses, which would further interfere with subsequent biomarkers screening for prediction the individual susceptibility to DILI. In addition, if a large number of differentially expressed genes (DEGs) existed in predose gene expression between the susceptible and resistant individuals, the validation of the potential of these genes to predict susceptibility to DILI is time-consuming and expensive. Thus, it is requiring a further screening method that could identify the drug responses-related DEGs in predose gene expression profiles in an unbiased manner, which could discover the more reliable biomarkers predictive of individual susceptibility to DILI.

**Fig 1:**
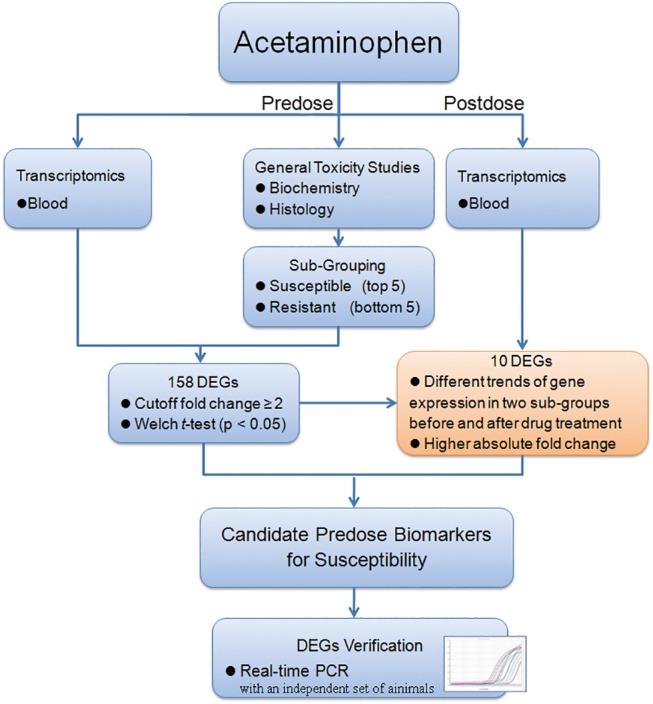
The overall research strategy and experimental design for this study.

Knowing that what should be overcome, we decided to test the hypothesis that combination of predose and postdose gene expression profiles of the susceptible and resistant individual animals contains sufficient information to allow the further screening of the DEGs in predose data, which can identify candidate gene biomarkers related to drug responses prior to drug treatment. Fig 1 shows the overall research strategy and experimental design for this study. Briefly, to assess our hypothesis, we chose the commonly used analgesic APAP for this investigation and compared the gene expression profiles in the blood collected prior to APAP administration with posterior to APAP treatment in susceptible and resistant Wistar rats. With this approach, we could relate the intrinsic individual variation more specific to DILI, providing an important clue for the determination of susceptible population to DILI.

## Materials and Methods

### Animals and ethics statement

Male Wistar rats (180−200 g, Silaike Co. Shanghai, China) were housed in an environmentally controlled room at 22 ± 1°C with a relative humidity of 50% ± 5%, and a light-dark cycle of 12 h each. Food and water were provided *ad libitum*. All experiments involving laboratory animals were approved and conducted in accordance to the guideline of the Institutional Animal Care and Use Committee (IACUC) of Zhejiang University School of Medicine. Surgical procedures were conducted under chloral hydrate (5% w/v, 0.6 mL/100 g body weight, Shanghai Qiangshun Chemical Reagent Co., Ltd., China) anesthesia, and all efforts were made to minimize suffering.

### Experimental design

To determine the intrinsic gene expression in blood for individual variation of APAP-induced liver injury (AILI), we analyzed the gene expression profiles in the blood collected from individual rats prior and posterior to APAP administration. In brief, the rats of first set were randomly divided into two groups, control group (n = 5) and APAP group (n = 35). The predose blood samples were collected via retro-orbital plexus from anesthetized animals into vacutainer-ethylene diamine tetraacetic acid (EDTA) tubes (BD Diagnostic Systems Sparks, USA) for RNA isolation. After a recovery period for 1 week, the rats were fasted for 12 h before once orally administrated with APAP (TCI (Shanghai) Development Co., Ltd., China, purity > 98%) at 1200 mg/kg in a 1% carboxymethyl cellulose sodium solution (China National Pharmaceutical Group Co., China). After drug administration, the rats were fasted for 2 h. The dose and treatment schedule were selected based on our preliminary experiments that induced the liver injury adequately. The animals were anesthetized with chloral hydrate (5% w/v, 0.6 mL/100 g body weight) at 24 h after APAP administration. The postdose blood samples were collected via inferior vena cava from anesthetized animals into vacutainer-EDTA tubes for RNA isolation. The livers were immediately removed from each animal and the excised samples from the left lateral liver lobe were used for histopathological examinations. Serum was collected from predose and postdose blood, and separated by centrifugation at 4000 rpm for 15 min at 4°C for biochemical analysis.

Then, based on ranking in the serum levels of alanine transaminase (ALT) after 24 h APAP administration, five most susceptible (top 5 in ALT levels) and five most resistant rats (bottom 5 in ALT levels) were selected as two sub-groups that showed susceptibility or resistance to AILI. Moreover, microarray analysis was performed to the precollected blood samples of these ten rats prior to APAP treatment, and the blood gene expressions in predose data were compared between these two sub-groups to identify the DEGs for individual variation. Subsequently, we detected the postdose gene expression profiles of these ten rats that could further screen the predose DEGs, which was aimed to relate intrinsic DEGs to drug responses for predicting the susceptibility of AILI. The details of the further screening method were shown in "Microarray data analysis" subsection. Finally, to investigate whether the DEGs obtained from the further screening might identify the susceptible animals, we performed the real-time PCR analysis to these genes with the blood samples precollected before APAP treatment from an independent test set of 37 rats (two groups, control group (n = 5) and APAP group (n = 32)). After a recovery period for 1 week, the rats were also treated with APAP as described above. Serum and liver samples were collected 24 h after the APAP treatment for biochemical analysis and histopathological examinations. The susceptibility to AILI based on the predicted results from real-time PCR was compared through analysis of serum biochemistry and liver histopathology after actual exposure to APAP. The details of prediction method were described in "mRNA quantitation by quantitative reverse transcriptase polymerase chain reaction analysis" subsection.

### Histopathology

The livers were collected 24 h after the APAP administration with 10% formalin. Then the tissues were embedded in paraffin, cut approximately 4 μm thick, and stained with hematoxylin and eosin (HE) for histological observation in a blind fashion.

### Serum biochemical analysis

The parameters of serum samples, including ALT, aspartate aminotransferase (AST), alkaline phosphatase (ALP), and total bilirubin (TBILI), were tested with a Roche COBASC 311 Automatic Analyzer.

### RNA extraction, purification, and microarray analysis

Within 24 h after collection, the total RNA was extracted from TRIZol Reagent (Invitrogen, USA) after lysing red blood cells (TIANGEN, China). The RNA purification and quality assessments were carried out as described previously [11]. Only RNA with RNA integrity numbers greater than 7.0 and 28S rRNA/18S rRNA more than 0.7 was used for microarray analysis. Affymetrix Rat Genome 230 2.0 chips were used according to manufacturer’s protocols as described in our previous study (n = 5) [11].

### Microarray data analysis

Gene expression data from the Affymetrix Rat Genome 230 2.0 chips were loaded into ArrayTrack^®^ (http://www.fda.gov/ScienceResearch/BioinformaticsTools/ArrayTrack) for data management, analysis, visualization, and interpretation as previously described [11]. In brief, raw microarray intensity data were normalized per chip to the same median intensity value of 1000. To exclude false DEGs due to low abundance transcripts, genes with normalized intensity less than 100 in all chips of predose gene expression data were excluded. The DEGs in predose gene expression data were selected using the Welch's t-test within ArrayTrack^®^ with cutoff values of *p* < 0.05 and absolute fold change (FC) ≥ 2 [12, 13]. For the further screening, the predose DEGs were divided into two categories based on the expression trends of the genes in the two sub-groups before and after APAP administration. One category was $\text{FC}\left(\frac{RA}{R}\right)\times FC\left(\frac{SA}{S}\right)$ < 0, where $\text{FC}\left(\frac{RA}{R}\right)$ means the fold change of a single gene's expression in resistant subset prior (R) and posterior (RA) to APAP administration; $\text{FC}\left(\frac{SA}{S}\right)$ indicated the fold change of a single gene's expression in susceptible subset before (S) and after (SA) APAP administration. Taken together, $\text{FC}\left(\frac{RA}{R}\right)\times FC\left(\frac{SA}{S}\right)$ < 0 reflected an opposite expression trend of a gene in the two sub-groups before and after APAP administration, i.e., the gene was upregulated (or downregulated) in susceptible group and downregulated (or upregulated) in resistant group after APAP treatment, compared with predose gene expression data. The other category was $\text{FC}\left(\frac{RA}{R}\right)\times FC\left(\frac{SA}{S}\right)$ > 0, which suggested a consistent expression trend of a gene in the two sub-groups before and after APAP administration, i.e., the gene was both upregulated or downregulated in susceptible and resistant groups after APAP treatment, compared with predose gene expression data. At last, these two sets of the genes were sorted with the absolute values of the change in descending order with two algorithms. When $\text{FC}\left(\frac{RA}{R}\right)\times FC\left(\frac{SA}{S}\right)$ < 0, we calculated the absolute values of $\text{FC}\left(\frac{RA}{R}\right)$ multiplied by $\text{FC}\left(\frac{SA}{S}\right)$; when $\text{FC}\left(\frac{RA}{R}\right)\times FC\left(\frac{SA}{S}\right)$ > 0, we counted the values of $\text{FC}\left(\frac{RA}{R}\right)$ divided by $\text{FC}\left(\frac{SA}{S}\right)$ or $\text{FC}\left(\frac{SA}{S}\right)$ divided by $\text{FC}\left(\frac{SA}{S}\right)$, ensuring that the larger values between $\text{FC}\left(\frac{RA}{R}\right)$ and $\text{FC}\left(\frac{SA}{S}\right)$ to be the numerator. Top five genes in each and total ten genes in these two-trend sets were chosen for further investigation. The entire set of microarray data was deposited in Gene Expression Omnibus (GEO) database with the number of GSE68065.

### mRNA quantitation by quantitative reverse transcriptase polymerase chain reaction analysis

To investigate whether the top 10 DEGs obtained from further screening might identify the susceptible animals, real-time quantitative reverse transcriptase polymerase chain reaction (qRT-PCR) was conducted with the blood samples precollected before APAP treatment from an independent set of 37 rats (two groups, control group (n = 5) and APAP group (n = 32)). With the genes selected from further screening, 8 rats with the lowest expression (the fourth quartile of total 32) and 8 rats with highest expression (the first quartile of total 32 animals) were allocated to predicted susceptible and resistant groups based on the results of microarray analysis and the risk allocation strategy described previously [14]. The susceptibility to AILI was compared between these two groups through analysis of serum biochemistry and histopathological examinations after actual exposure to APAP. The qRT-PCR reactions were carried out as previously described [11, 15]. The sequences for the primer pairs used are listed in Supplementary S1 Table. qRT-PCR data for each gene product were normalized to the levels of 18S rRNA transcript. The ratio of the target gene to the housekeeping gene (18S rRNA) was calculated and expressed as 2^-ΔCt^. This ratio was then used to evaluate the expression level of the target gene of each animal. Based on a previous study [9], to determine the fold changes in expressions among animals, the normalized gene expression of the target genes was divided by the normalized expression of the same gene in the sample with the lowest level of the normalized gene expression of the target genes, expressed as 2^-ΔΔCt^. In addition, the expression levels of the candidate genes were also detected in liver tissues by qRT-PCR after exposure to APAP. The data were normalized to the levels of 18S rRNA transcript and expressed as 2^-ΔCt^.

### Statistical analyses

Statistical differences between susceptible and resistant groups were determined by one-way ANOVA, followed by Student’s t-test. For all comparisons, a *p* value < 0.05 was considered to be statistically significant.

## Results

### Individual difference in the liver injury after APAP administration

**Fig 2:**
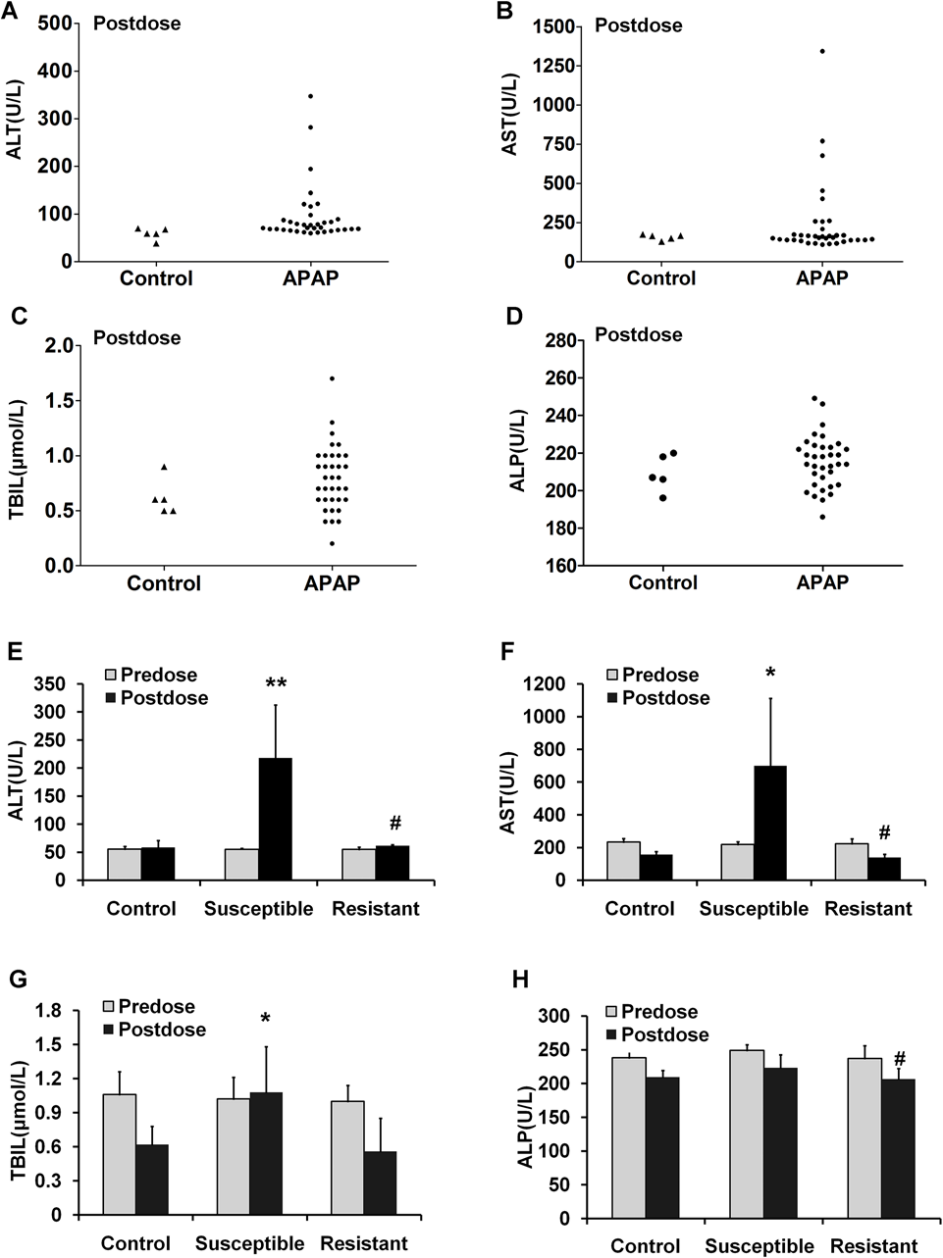
Serum biochemical analysis prior and posterior to APAP administration. (A-D) The scatter plots of ALT (A), AST (B), ALP (C), and TBILI (D) levels of each animal in the first set. (E-H) Comparison of the levels of ALT (E), AST (F), ALP (G), and TBILI (H) was performed in control, susceptible, and resistant sub-groups before and after APAP treatment in the first set (n = 5). Values are represented as mean ± SD. * *p* < 0.05, ** *p* < 0.01 compared with vehicle control. ^#^ *p* < 0.05 compared with susceptible sub-group. Con, control group; Sus, susceptible sub-group; Res, resistant sub-group.

**Fig 3:**
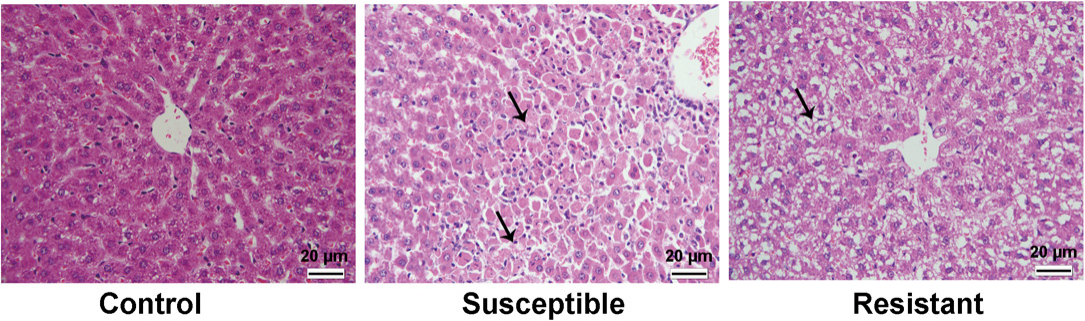
Representative images of rat livers after APAP administration with histopathological examinations of HE stain. The rats in control group showed normal morphology of liver. Susceptible group exhibited massive hepatic injury including necrosis and inflammatory cells infiltration as arrows indicated, whereas the rat in resistant group showed hydropic degeneration of hepatocytes (arrow). The magnification used was 400×. The scale bar is 20 μm.

In order to predict the individual susceptibility to AILI, a representative outbred strain Wistar rat was used in this study. As expected, severe liver injury was induced in a part of rats after APAP administration, and inter-subject variation in AILI was observed as evidenced by the different extent of elevation in the levels of serum ALT, AST, TBILI, and ALP parameters (Fig 2A–2D). On the basis of the ranking in serum ALT, APAP-treated rats were divided into two sub-groups, susceptible (top five in ALT levels) and resistant groups (bottom five in ALT levels). As shown in Fig 2E–2H, no significance was detected among control, susceptible and resistant groups in these serum parameters before APAP treatment, but markedly higher levels of serum ALT, AST, and TBILI were observed in the susceptible group compared with control group after APAP treatment, and significantly higher levels of ALT and AST were noted in the susceptible group compared with resistant group posterior to APAP administration. The results of histological examinations such as necrosis and inflammatory cell infiltration in the livers of all five susceptible rats also supported the results obtained from the serum biochemical assays, whereas only hydropic degeneration of hepatocytes was observed in the livers of the five resistant rats (Fig 3).

### Gene expression analysis

We performed microarray analysis using the blood samples collected before and after APAP treatment from the five most susceptible and five most resistant rats. First, the predose gene expressions between these two sub-groups were compared to identify the DEGs for individual variation before drug administration. As a results, 158 genes were identified to be statistically different between the two sub-groups (*p* < 0.05 and fold change ≥ 2). To further relate these 158 DEGs to drug responses, we screened them with postdose gene expression data, which was based on the expression trends of these genes in the two sub-groups before and after APAP treatment as described in ‘‘Materials and Methods” section. Briefly, if a gene from the 158 DEGs has the opposite expression trend in susceptible and resistant groups after APAP treatment, it would be more likely to be a candidate biomarker for prediction the susceptibility to AILI, such as downregulated in susceptible group and upregulated in resistant group after APAP treatment compared with the predose expression data. Simultaneously, if a gene from the 158 DEGs has the consistent expression trend in susceptible and resistant groups after APAP treatment and large difference was observed in the fold changes in these two sub-groups, it would be also more likely to be a candidate biomarker for prediction the susceptibility to AILI. Thus, we used two algorithms to calculate the changes of these two situations as described in ‘‘Materials and Methods” section. After ranking, top five genes in each trend set with larger absolute values in the algorithms were chosen as candidate predose biomarkers for detecting individuals susceptibility to AILI, i.e., total ten genes in these two-trend sets. Among them, *My17*, *Bmp2*, *Mmp12*, *Gprin1*, and *Sox11* had opposite expression trend in susceptible and resistant groups after APAP treatments compared with predose data, whereas *Rpgrip1*, *Hrg*, *Incenp*, *Sbf1*, and *S100b* had the consistent expression trend in these two sub-groups (Table 1).

**Table 1:** The expressions of the 10 genes in predose and postdose gene expression profiles which were identified as potential gene biomarkers to distinguish between susceptible and resistant rats to AILI.

Gene_id_mfr	Genebankacc	Gene name	Locusid	$\text{FC}\left(\frac{RA}{R}\right)$	$\text{FC}\left(\frac{SA}{S}\right)$	Absolute values of $\text{FC}\left(\frac{RA}{R}\right)\times$ $\text{FC}\left(\frac{SA}{S}\right)$	Values of $\text{FC}\left(\frac{RA}{R}\right)÷$ $\text{FC}\left(\frac{SA}{S}\right)$ or $\text{FC}\left(\frac{SA}{S}\right)÷$ $\text{FC}\left(\frac{RA}{R}\right)$
1371315_at	AA891242	Myl7	289759	5.22	-3.05	15.92	
1368945_at	NM_017178	Bmp2	29373	1.19	-7.08	8.43	
1368530_at	NM_053963	Mmp12	117033	-1.61	4.81	7.74	
1375050_at	BI294558	Gprin1	364676	-1.28	5.63	7.21	
1387275_at	NM_053349	Sox11	84046	1.61	-4.36	7.02	
1375407_at	BG374229	Rpgrip1	305850	-5.95	-1.29		4.61
1368583_a_at	NM_133428	Hrg	171016	1.03	3.46		3.36
1386903_at	NM_013191	S100b	25742	3.25	1.02		3.19
1378669_at	AI706871	Incenp	293733	1.11	3.40		3.06
1377174_at	AW434982	Sbf1	300147	1.02	2.99		2.93

### Prediction of the individuals susceptibility to AILI by real-time PCR analysis

**Fig 4:**
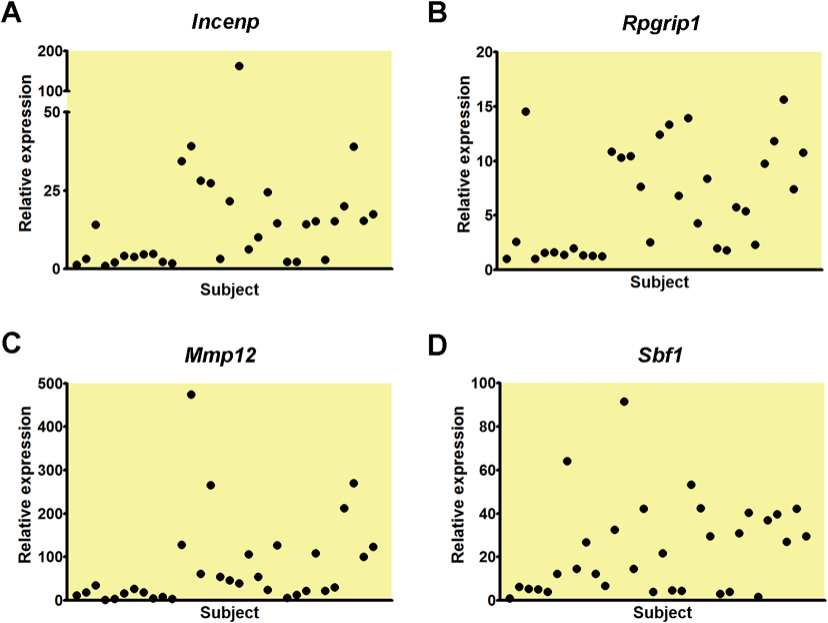
The real-time PCR analysis of the gene expression of the selected blood genes prior to APAP administration in a new test set of rats. (A-D) Scatter plots for the individual expression levels of *Incenp* (A), *Rpgrip1* (B), *Mmp12* (C), and *Sbf1*(D) of the new set of 32 rats in APAP group.

Finally, to investigate whether the DEGs obtained from further screening might identify the susceptible animals, we conducted the real-time PCR analysis with the blood samples precollected before APAP treatment from an independent set of 37 rats (two groups, control group (n = 5) and APAP group (n = 32)). With the 10 genes selected from further screening, the expression levels of four genes had a inter-individual variation in APAP group before drug treatment (Fig 4). To evaluate whether the individual expression levels of these four genes, *Incenp*, *Rpgrip1*, *Mmp12*, and *Sbf1*, might predict the susceptible individuals to AILI indeed, the rats were subdivided to four quartiles based on the predose blood gene expression levels. The 8 rats had lowest gene expression levels of 32 rats, was predicted to susceptible group (first quartile), and the 8 rats had highest gene expression levels of 32 rats, was predicted to resistant group (fourth quartile), which was based on the results of the first set.

**Fig 5:**
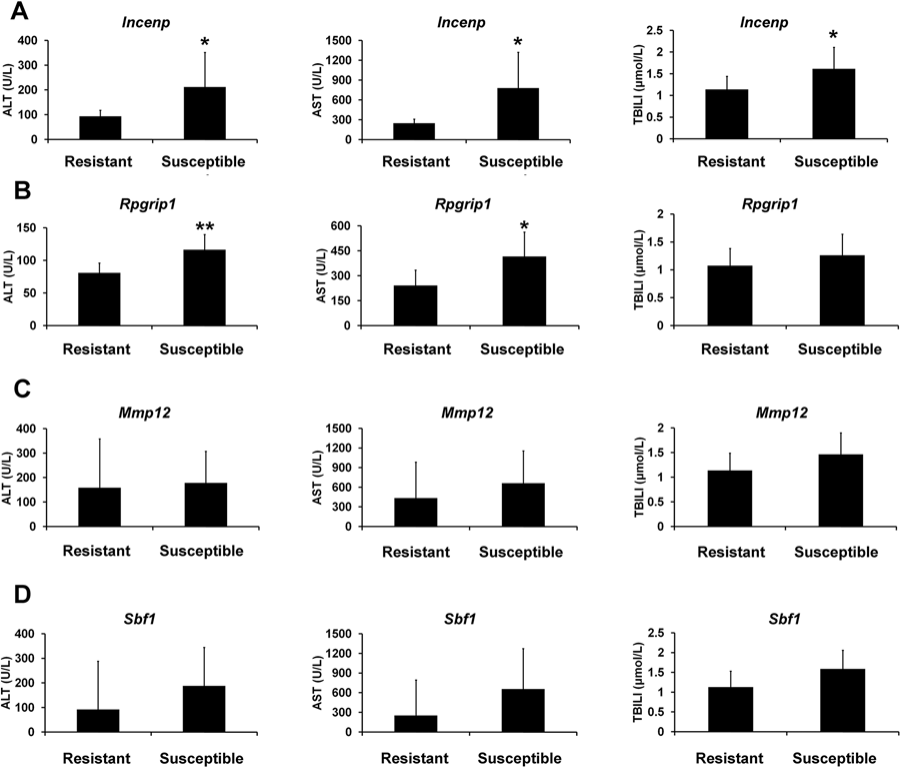
Comparison of the serum ALT, AST, and TBILI levels between predicted susceptible and resistant groups selected by predose expression levels of *Incenp*, *Rpgrip1*, *Mmp12*, and *Sbf1* in precollected blood samples prior to APAP administration in the new set of 32 rats. The 8 rats had lowest gene expression levels of 32 rats were predicted to susceptible group, whereas 8 rats had highest gene expression levels of 32 rats were predicted to resistant group. (A) *Incenp*, (B) *Rpgrip1*, (C) *Mmp12*, and (D) *Sbf1*. Values are represented as mean ± SD. * *p* < 0.05, ** *p* < 0.01 compared with resistant group.

**Fig 6:**
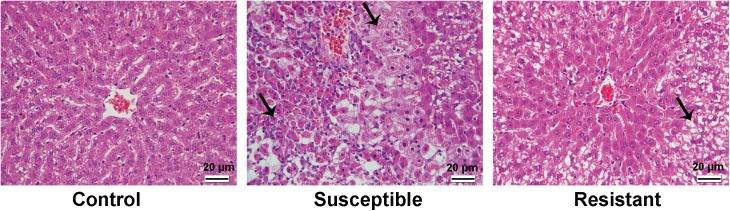
Representative images of histopathological examinations of the rat livers between predicted susceptible and predicted resistant groups after APAP administration in the new set of 32 rats with HE stain. Similar to the results of first set, the rats in control group showed normal appearance of liver, whereas predicted susceptible group exhibited serious liver injury including necrosis and massive inflammatory cells infiltration as arrows indicated, and the rat in predicted resistant group showed hydropic degeneration of hepatocytes (arrows). The magnification used was 400×. The scale bar is 20 μm.

**Fig 7:**
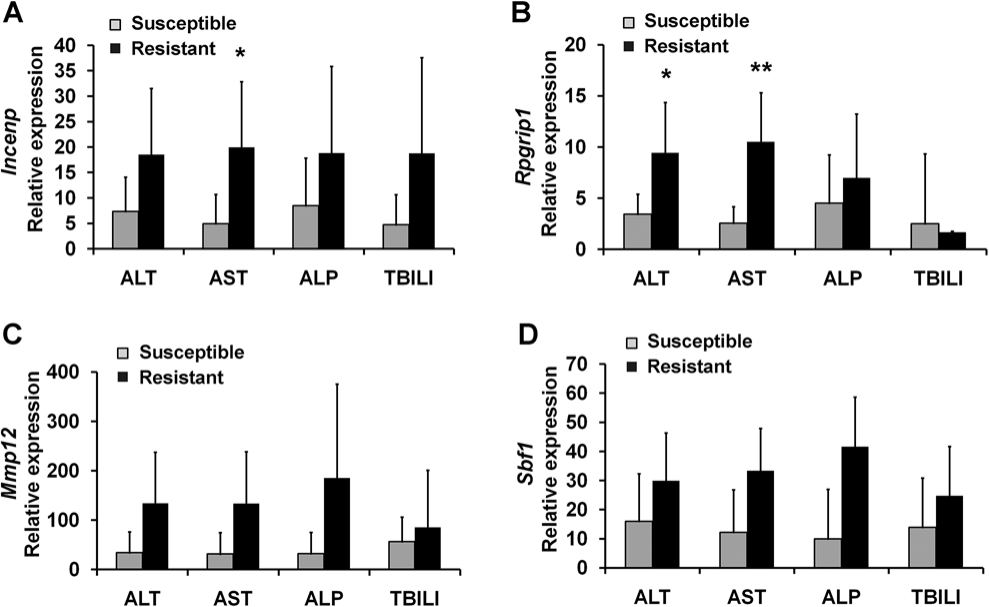
The expression levels of *Incenp*, *Rpgrip1*, *Mmp12*, and *Sbf1* between the five most susceptible rats and the five most resistant rats in precollected blood samples prior to APAP administration in the new set of 32 rats. The five most susceptible rats and the five most resistant rats were selected based on ranking in the ALT, AST, ALP, and TBILI levels after APAP treatment, respectively. The expression levels of individual animals of these genes were calculated as described above and in "Materials and methods". (A) *Incenp*, (B) *Rpgrip1*, (C) *Mmp12*, and (D) *Sbf1*. Values are represented as mean ± SD. * *p* < 0.05, ** *p* < 0.01 compared with susceptible group.

Then, by measuring the levels of serum biochemical parameters after APAP treatment, the actual susceptibility to AILI was determined. As a result, the 8 predicted susceptible rats with lower expression levels in the two genes, *Incenp* and *Rpgrip1*, showed significant higher ALT, AST and/or TBILI levels than predicted resistant animals, while for the other two genes, *Mmp12* and *Sbf1*, the trend was the same, but no significant differences were detected between these two predicted sub-groups (Fig 5, the data of ALP were not shown, because there were no difference detected between the two sub-groups). The results of histopathological examinations also showed that necrosis and inflammatory cell infiltration were detected in all the rats of predicted susceptible group, whereas hydropic degeneration of hepatocytes were observed in all the rats of predicted resistant group (Fig 6). Furthermore, based on the levels of serum biochemical parameters, ALT, AST, ALP, and TBILI, five most susceptible and the five most resistant rats were selected, respectively. As shown in Fig 7, the expression levels of the four genes showed differences between the two sub-groups in the selected rats based on the serum biochemical parameters, and the susceptible rats had the lower gene expression levels than resistant rats, especially selected by ALT and/or AST in *Incenp* and *Rpgrip1*, which was in good agreement with microarray and predicted results.

**Fig 8:**
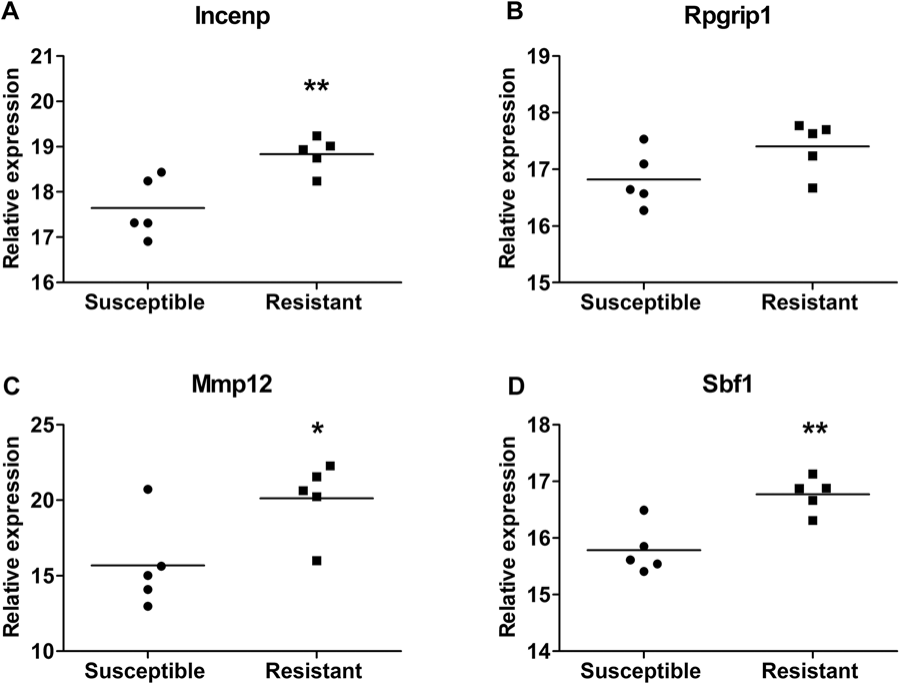
The expression levels of *Incenp*, *Rpgrip1*, *Mmp12*, and *Sbf1* in the liver tissues of the 5 most resistant and 5 most susceptible rats after APAP administration in the new set of 32 animals. The 5 most resistant and 5 most susceptible rats were selected by ALT levels. (A) *Incenp*, (B) *Rpgrip1*, (C) *Mmp12*, and (D) *Sbf1*. The relative expression levels were calculated by the ratio of the target gene to the housekeeping gene (18S rRNA) as 2^-ΔCt^. Lines are represented as the mean values. * *p* < 0.05, ** *p* < 0.01 compared with susceptible group.

Additionally, the expression levels of these four genes in liver tissues were also detected in the 5 most resistant and 5 most susceptible rats selected by ALT levels in the new set animals after APAP administration. As the results, the four genes (*Incenp*, *Rpgrip1*, *Mmp12*, and *Sbf1*) were all higher expressed in the livers of resistant rats compared with susceptible animals after drug exposure (Fig 8, although no significance was detected in *Rpgrip1*), which had the same trend as blood samples before drug treatment. It indicated that these genes were related to the susceptibility of AILI.

## Discussion

As DILI is a critical problem in the world with new agents added every year, there is increasing researches aimed at better understanding the mechanisms and individual susceptibility of DILI [16]. The difference of inter-subject susceptibility is likely to arise from complex interactions involved in environmental and genetic factors. It demonstrated that predose global gene expression profiles of biofluids reflect inter-subject variation with respect to both genomic and environmental influences, and hepatotoxicants can produce compound-specific changes in postdose transcriptome of peripheral blood [17]. Thus, in this study, we combined the predose and postdose gene expression profiles of blood samples to further screen the DEGs in predose data between susceptible and resistant rats to AILI, which aimed to relate the intrinsic individual variation more specific to AILI and identify more reliable gene biomarkers for prediction individuals susceptibility prior to drug treatment. Although a few studies suggested that rats are much more resistant to APAP hepatotoxicity than mice and are not a valid model for mechanistic studies [18–20], many researchers have continued to use this species for exploring the potentially hepatoprotective compounds and the mechanisms of AILI in recent years [21–25]. In this study, rats were chosen as model animals rather than mice since they could provide enough blood for both transcriptomics and biochemistry analyses in the predose and postdose periods. After further validation, we suggested that the expression levels of *Incenp* and *Rpgrip1* in the blood might identify the susceptible individuals to AILI before actual exposure to APAP.

APAP is a drug caused approximately 50% cases of acute liver failure in the United States and Great Britain [26], thus, it was chosen as a model drug in this study. Moreover, inter-individual variation in response to APAP is a regulatory concern and a hot topic in DILI. Recently, Liu et al. identified that betaine-homocysteine methyltransferase 2 can affect susceptibility to AILI in mice using an integrative genetic, transcriptional, and metabolomic analysis in multiple inbred mouse strains [27]. Harrill et al. also demonstrated that utilization of inbred mouse strains is a valuable tool for evaluating individuals susceptibility to AILI by genetic polymorphisms analysis or global gene expression profiles [28, 29]. Welch et al. suggested that proteomic analysis can identify the potential susceptibility factors in AILI [30]. However, these studies have examined the influential factors of inter-subject susceptibility to AILI after drug treatment, and therefore, possibly many indicated biomarkers could be the result of secondary responses to pathophysiological changes by APAP. The ability of systems biology approaches to predict susceptibility to DILI prior to drug treatment has been demonstrated both in human and rodents in the recently studies. For instance, urine metabolite profiles obtained before the start of treatment were sufficient to distinguish which of the subjects would develop liver injury after given a single toxic-threshold dose of APAP in outbred strain rats [31], whereas urine metabolite profiles obtained shortly after the start of APAP treatment in maximum recommended daily dose, but prior to ALT elevation, could distinguish responders from nonresponders in human [32]; Yun et al. have recently devised a novel method that examines the innate gene expressions of biofluids and biopsy tissues prior to drug treatment to predict the susceptibility of DILI, including AILI [7–10]. Particularly, they identified protein kinase A inhibitor α as a potential biomarkers for screening the susceptibility of AILI [10]. Nevertheless, one critical disadvantage existed in these studies is that a number of inter-individual variation in predose data before drug treatment are not associated with drug responses, which would further interfere with subsequent biomarkers screening for prediction the individual susceptibility to DILI. Besides, the large number of inter-individual variation existed in predose data would hinder the efficiency of further validation. Thus, in this study, we demonstrated that combination of predose and postdose gene expression profiles contains sufficient information to allow the further screening of the inter-individual variation in predose data, which deeply considered the changes of signal gene's expression both before and after drug treatment in susceptible and resistant animals and could identify more reliable biomarkers related to drug responses to predict individuals susceptibility of AILI.

In this study, 158 genes were found to be statistically different in their expression levels between susceptible and resistant sub-groups prior to APAP administration. After further screening with postdose gene expression data by the two formulas mentioned above, 10 genes would be the more reliable candidate biomarkers. Of these genes, the expression of *Incenp* and *Rpgrip1* was verified to closely relate to individual susceptibility to AILI with an independent set of animals. *Incenp* encodes inner centromere protein. The latter forming a complex with Aurora-B and Survivin, regulates the stability of bipolar spindle-kinetochore attachment in mitosis and chromosome segregation and cytokinesis [33]. Overexpression of *Incenp* would be suggestive of an increase in a cell population with chromosomal instability [34]. Downregulation of *Incenp* was observed in the mice spleens after APAP treatment, indicating anti-proliferative effects in immune cells [35], whereas Incenp-immunoreactive cells were significantly increased in livers after APAP treatment [34]. In this study, the expression levels of *Incenp* were higher in resistant rats than those of susceptible rats in predose data. In this regard, we inferred that rats with innately higher expression of *Incenp* might be resistant to AILI, because these rats may have more possibility to induce cell proliferation and tissue regeneration. Supporting this in part, several proteins involved in cell proliferation and tissue regeneration were more highly expressed in the livers of resistant mice compared with susceptible mice in AILI after APAP treatment [30]. The expression of *Rpgrip1* in blood was also observed to be innately higher in the resistant rats to AILI. *Rpgrip1* was discovered in the retina, but multiple splice variants and protein isoforms were found in various tissues in the rodents and human, including liver [36, 37]. The changes of *Rpgrip1* expression are associated with tissue growth factors [37]. Thus, we speculated that increased resistance to AILI in the rats with innately higher expression of *Rpgrip1* might be the result of enhancing tissue growth ability to APAP-induced tissue damages.

Although there was no significant difference in the results of *Mmp12* and *Sbf1* in the validation experiments between two sub-groups, the expression of *Mmp12* and *Sbf1* in blood was also detected to be innately higher in the resistant rats to AILI. *Mmp12* encodes a protein of the matrix metalloproteinase family which is involved in normal physiological processes as reproduction and tissue remodeling, as well as in disease processes [38]. Increases in MMP-9, -10, and -12 proteins were observed in liver after treatment with hepatotoxin dimethylnitrosamine, which were associated with tissue repair, metastasis, and tissue remodeling [38]. Other MMPs also increased in the liver in response to carbon tetrachloride (CCl_4_) administration, relating to inflammatory reactions [39], as well as APAP treatment, which were associated with hepatocellular damage and hepatic microcirculatory dysfunction [40]. Moreover, MMP12 haplotype may play a critical role in susceptibility to severe airway and lung injury in children with chronic bronchitis and recurrent pneumonia [41]. Therefore, we inferred that increased susceptibility to AILI in the rats with innately lower expression of *Mmp12* might be the result of lacking tissue repair and remodeling ability to APAP-induced tissue damages. *Sbf1* encodes SET binding factor 1 which is a pseudo-phosphatase related to the myotubularin family of dual specificity phosphatases. Although there were no reports about the relationship between *Sbf1* and liver, this gene has been implicated in cellular growth and differentiation [42]. In this regard, innately higher expression of *Sbf1* in resistant rats might be increased the tolerance to AILI though cellular growth pathway.

Taken together, these four gene biomarkers were all closely related to cell proliferation and tissue repair functions, which were innately higher expressed in resistant rats to AILI. Moreover, the expression levels of these four genes were all higher expressed in the livers of resistant rats compared with susceptible animals after APAP treatment, indicating they were indeed related to the susceptibility of AILI. Thus, we believed that rats with higher ability of cell proliferation and tissue repair prior to drug treatment might be more likely resistant to AILI. Consistently, it has been demonstrated that the time of onset of tissue repair determines the extent of liver injury, and inter-individual differences in the magnitude of tissue repair may contribute significantly to individual susceptibility to DILI [43]. To clarify the results of this study further, how inter-individual differences in the magnitude of tissue repair influence the individual susceptibility to AILI should be elucidated such as *in vivo* siRNA experiments to these genes. Additionally, there was a situation should be indicated. Some rats with high expression in one or more than one of the four genes prior to drug treatment but exhibited mild or moderate susceptibility to AILI (data not shown). This might be from the contribution of other unidentified factors, which also observed in the susceptibility of CCl_4_-induced hepatotoxicity [9].

## Conclusion

In this study, we first proposed a method for the further screening of the intrinsic individual variation in the predose gene expression profiles, which can identify more reliable candidate biomarkers related to drug responses for detecting individuals susceptibility before drug treatment, and this method is also suitable to other system biological approaches, such as proteomics and metabonomics. More importantly, although further studies should be conducted with other hepatotoxicants or species to validate this approach, this study has shown the potential applicability of combination of blood gene expression prior and posterior to drug treatment as a novel and practical method to discover reliable biomarkers for prediction the susceptible population to DILI. However, the contribution of drug metabolism to the susceptibility of AILI was not addressed in this study, such as the individual differences in the formation of reactive intermediate N-acetyl-p-benzoquinone imine (NAPQI) by cytochrome P450 enzymes. The excess NAPQI is a leading cause of AILI. Despite this, our results suggest that further studies are needed on the functions of the genes controlling tissue repair in the susceptibility of AILI."

## Supporting Information

S1 Table The gene-specific primers used for qRT-PCR. (DOC)
