# Supplementary material for: Predose and Postdose Blood Gene Expression Profiles Identify the Individuals Susceptible to Acetaminophen-Induced Liver Injury in Rats
Source: PLoS One. 2015 Oct 29;10(10):e0141750. doi: 10.1371/journal.pone.0141750 (PMC4626237; doi:10.1371/journal.pone.0141750)
Supplement: S1 Table — (DOC) [file pone.0141750.s001.doc]

**Supporting Information**

S1 Table. Gene-specific primers used for qRT-PCR.

| **Gene symbol** | **NCBI Gene ID** | **Forward primer (5' −> 3')** | **Reverse primer (5' −> 3')** |
| --- | --- | --- | --- |
| Bmp2 | 29373 | ACGAGAAAAGCGTCAAGC | TCATTCCACCCCACATCA |
| Gprin1 | 364676 | AAAGAGCAGCCACAACCG | GATGCAGAGTCAGCCTTCC |
| Hrg | 171016 | CTCAAGGGTCAGTATCATCG | AGTGGAGGGAGTCGGTAG |
| Incenp | 293733 | GCCTCTGCTCGCATCATTT | CTTGCTCCTCTACTGCCTCCT |
| Mmp12 | 117033 | CCCATCCTTGACAAATCC | GCCTCCACCAGAAGAACC |
| Myl7 | 289759 | ACAAGTTCTCTCCTGCTG | ACTCCTCTTTCTCATCCC |
| Rpgrip1 | 305850 | AGTTCATCTCCGTTGGTTC | GTATGTGCCGTTCGTTATT |
| Sbf1 | 300147 | CCTGTCATTCGCTTCCAT | TGCCCTCCAACACTTTCAT |
| Sox11 | 84046 | GGAAACGGTGATGAAATGTTG | ACGATAAAGGAAGGGAAGAGTG |
| S100b | 25742 | AGACCTATCACCCACACC | ATCTAATCGTTCCACCAG |
| Rn18s | 100861533 | AAACGGCTACCACATCCAAG | TTGCCCTCCAATGGATCCT |
